# Extra-Adrenal Myelolipoma: A Rare Entity in Paediatric Age Group

**Published:** 2013-10-07

**Authors:** Chandrashekhar A Sohoni

**Affiliations:** Medicos Clinic, Koregaon Park, Pune, Maharashtra, India.

**Keywords:** Extra-adrenal myelolipoma, Pain in abdomen, Child

## Abstract

Extra-adrenal myelolipoma is a well-described entity in adult population, however it is extremely rare in paediatric age group. An unusual case of intra-peritoneal extra-adrenal myelolipoma in an 8-year-old child is presented here. The lesion was incidentally detected while evaluating the patient for spasmodic abdominal pain. Ultrasonography followed by CT scan and MRI imaging suggested the diagnosis which was confirmed by histopathology. A non-surgical approach was adopted and there was no progression of the lesion on follow-up imaging.

## INTRODUCTION

Extra-adrenal myelolipoma is a rare benign tumour with only about 50 cases reported previously.[1] Histologically, myelolipomas consist of haematopoietic elements and mature adipose tissue. Cases of extra-adrenal myelolipomas have been described previously only in middle aged and elderly individuals. Extra-adrenal myelolipomas are exceedingly rare in paediatric age-group.[2]

## CASE REPORT

An 8-year old girl presented with spasmodic pain in abdomen for one week. The pain was not localised to one particular region of the abdomen. It was not associated with fever, vomiting, diarrhoea or constipation. There was no significant past medical or surgical history. The general physical examination revealed mild tenderness on palpation in the right hypochondrium. Rest of the general and systemic examination was unremarkable. Ultrasonography of abdomen revealed an echogenic mass lesion in the right subhepatic region measuring 3.2 x 2.0 cm. Short term use of dicyclomine relieved the spasmodic abdominal pain. Further evaluation of the incidentally noted abnormality with computerised tomography (CT) scan revealed a well-defined intraperitoneal mass lesion in the right subhepatic region which contained areas of fat density interspersed with areas of soft-tissue density higher than fat. No evidence of calcification was seen within the lesion. Post-contrast CT scan revealed mild enhancement of the capsule of the lesion and the intrinsic areas of soft-tissue density (Fig. 1). No invasion of adjacent structures was seen. Magnetic Resonance Imaging (MRI) was performed out of academic interest and confirmed the presence of fat along with some other soft tissues within the lesion on T2W fat suppressed images (Fig. 2). The laboratory investigations were within normal limits. A CT guided biopsy of the lesion was performed. Histopathological analysis revealed presence of haematopoietic cells in various stages of maturation admixed with mature adipose tissue suggestive of myelolipoma. In view of the benign nature of the lesion, absence of any attributable symptoms and reluctance of parents for any surgical intervention the lesion was not operated upon. Follow-up ultrasonography at 6 months and 1 year did not reveal any significant change in the size and appearance of the lesion.

**Figure:**
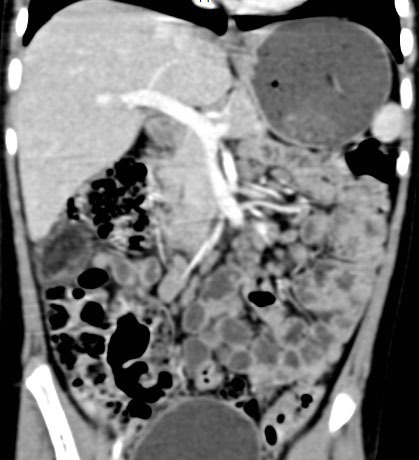
Figure 1: Presence of fatty component along with enhancing soft tissue is seen within the well-defined, encapsulated mass lesion on CT scan.

**Figure:**
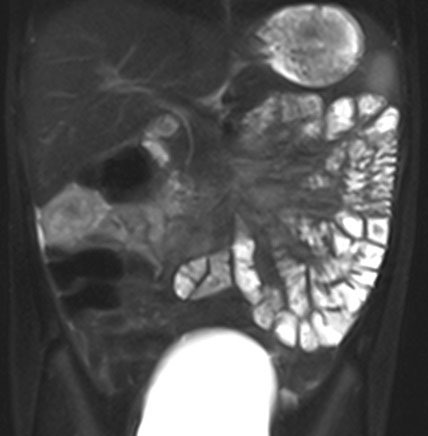
Figure 2: The fat suppressed T2W MRI sequence confirms the presence of fat within the lesion as shown by the arrow.

## DISCUSSION

Extra-adrenal myelolipoma is an uncommon but well-described entity in medical literature. It is being diagnosed with increasing frequency because of the wide availability and use of imaging modalities. The usual locations for extra-adrenal myelolipomas are retroperitoneum, pre-sacral region and pelvis. [3] The median age for diagnosis of extra-adrenal myelolipoma is 66.5 years.[4] The unique features of the case described above are the age of presentation and the location of the lesion. To the best of our knowledge, only one case of extra-adrenal myelolipoma has been described previously in paediatric age group.[2]

The aetiology is not known but endocrine disorders and chronic diseases are believed to play a role.[5] In our case, no such underlying co-morbidity was found. Many patients are asymptomatic at diagnosis, while some may present with non-specific symptoms such as abdominal discomfort, nausea and vomiting. Surgical treatment of myelolipoma is indicated only if the patient is symptomatic or the tumour is causing mass effect. In asymptomatic patients, the lesion may be monitored by periodic imaging. In our patient, the spasmodic pain was unlikely to be related to the lesion, because the pain was not localised to right hypochondrium and short term use of an antispasmodic relieved it.

Though specific pre-operative diagnosis of extra-adrenal myelolipoma is difficult, imaging can be suggestive.[6] The fatty component of the lesion appears hyperechoic on ultrasonography, whereas the myeloid component appears hypoechoic. CT scan is more specific for the presence of fat within the lesion. The myeloid component shows higher attenuation as compared to fat and may also show enhancement on post-contrast CT study. Extra-adrenal myeloliomas are well-encapsulated lesions and may show capsular enhancement on post-contrast CT, as seen in our patient. Presence of fat within the lesion is confirmed on T1 weighted and fat-suppressed MRI sequences. The radiological differential diagnosis includes lipoma, dermoid, liposarcoma, myolipoma, angiomyolipoma and extramedullary haemopoiesis.[5] Core biopsy of the lesion establishes definitive diagnosis, thus preventing unnecessary surgical intervention.[6]

## Footnotes

**Source of Support:** Nil

**Conflict of Interest:** None declared

